# Chinese Herbal Medicine Bushen Qinggan Formula for Blood Pressure Variability and Endothelial Injury in Hypertensive Patients: A Randomized Controlled Pilot Clinical Trial

**DOI:** 10.1155/2014/804171

**Published:** 2014-06-16

**Authors:** Chunxiao Wu, Jingchun Zhang, Yingke Zhao, Jing Chen, Yue Liu

**Affiliations:** ^1^Cardiovascular Diseases Center, Xiyuan Hospital of China Academy of Chinese Medical Sciences, Beijing 100091, China; ^2^China Heart Institute of Chinese Medicine, China Academy of Chinese Medical Sciences, Beijing 100091, China

## Abstract

*Background.* Blood pressure variability (BPV) independent of average blood pressure is related to cardiovascular damage. Meanwhile, BPV is also associated with measures of endothelial injury. Decoction, a traditional used form of Traditional Chinese Medicine (TCM), is inconvenient to prepare, carry, and store. Dispensing granules is now developing as an alternative to decoction, but the evidence supporting its clinical efficacy the same as decoction remains unclear. *Objective.* To examine the therapeutic effects on mean blood pressure (MBP), blood pressure variability, and endothelial function by giving Bushen Qinggan Formula, a compound Chinese Herbal Medicine and also to evaluate the difference in efficacy between decoction and granule. *Methods.* A total of 150 patients with hypertension were enrolled and randomly assigned to receive the placebo, Bushen Qinggan decoction, or Bushen Qinggan granule in addition to the standard medications (amlodipine-5 mg/d) for the treatment of essential hypertension (EH). The outcome was the reduction in the MBP and BPV and also included changes in the endothelial markers including endothelin-1 (ET-1) and nitric oxide (NO) after 8 weeks of treatment. *Results.* Compared with the control group, the Bushen Qinggan decoction and granule groups had significant improvement (*P* < 0.01) in BPV and endothelial founction. The level of BPV and endothelial function between decoction and granule group had no significant difference (*P* > 0.05). *Conclusion.* Based on the standard treatment, Bushen Qinggan Formula further improved BPV and endothelial function. The efficacy of Bushen Qinggan decoction and granule is similar in improving BPV and endothelial function. However, no significant antihypertensive effects could be demonstrated.

## 1. Introduction

In developed countries, during one's lifetime, the risk of becoming hypertensive exceeds 90% [1]. Now, data from national surveys show that hypertension is also very common in China, almost 1 in 5 adults diagnosed with high blood pressure (BP) [2]. The morbidity of hypertension has been increasing in China for decades; however, the prevalence, awareness, management, and control of hypertension from the current study are still much lower than those reported by the NHANES (National Health and Nutrition Examination surveys), 1999–2010 [3]. BP is a continuous, consistent, and independent risk factor for cardiovascular disease and stroke [4]. Stroke is also the major complication of hypertension in the Chinese population [2]. Two-thirds of strokes and half of coronary disease can be attributed to nonoptimum BP [5]; therefore, the final purpose of controlling of hypertension is to reduce the cases of events [6]. A cost-effective approach should be put in practice to achieve these goals in resource-limited settings in China.

Hypertension is one of the most prevalent vascular risk factor; the mechanisms contributing to the increased end-organ damages and vascular events risk as yet are unclear [7]. Nevertheless, it has been suggested that increased variability of blood flow induces the augmented mechanical stress. The pressure placed on the blood vessels may induce the endothelial dysfunction [8]. Blood pressure variability (BPV) plays a vital role in the progression of end-organ damage and in triggering of vascular events. Two recent studies, Pressioni Arteriose Monitorate E Loro Associazioni (PAMELA) and the Ohasama, both reported that measures of BPV within a 24-hour period were independent predictors of cardiovascular mortality [9, 10]. In addition, antihypertensive drugs, which decrease both BPV and mean blood pressure, more effectively reduce the risk of target organ damage [11]. Meanwhile, BPV is also associated with measures of endothelial injury and endothelial function [12].

When considering that impaired endothelial function has been strongly associated with cardiovascular morbidity and mortality, the previously observed associations between high BPV and increased cardiovascular risk could be mediated via the impairment of endothelial function. Our preliminary study shows that Chinese herbal medicine “Bushen Qinggan Formula” improved endothelial injury in patients with high blood pressure [13]. The purpose of this study, therefore, was to investigate that whether Bushen Qinggan Formula improves both endothelial function and ambulatory BPV.

Decoction, a traditional and commonly used form of Traditional Chinese Medicine (TCM), is prepared by decocting different kinds of medicinal herbs together [14]. While the benefits of decoction are apparent, it is more beneficial for absorption and having higher bioavailability, and the compliance of the patient also needs to be assessed. Most hypertensive patients need lifelong medication. Similarly, Chinese medicine treatment can take a very long time. The preparation of decoction is so completed that a part of patients failed to sustain the course of treatment. That is why it is very difficult for patients to decoct TCM daily until a significant effect can be seen. Granules are much more convenient; they only need to dissolve in boiling water. If granules really can replace decoction, the compliance of patients definitely will be improved.

Dispensing granule is the granule of individual herb. Dispensing granules now are developing as an alternative to decoction, prescribed by traditional medicine practitioners in China, Japan, Korea, Singapore, even United States, and some European countries. The popularization of the dispensing granules may be because that one of the most obvious inconveniences of TCM is the preparation. Therefore, this research group decided to compare the effect of granule and decoction.

Chinese herbal formulas are known to have an advantage with regard to prevent and treat target organ damage (TOD) [15]. Researches also show that Chinese medicine has some protective function, including both improvement of BPV and endothelial function [16, 17]. Bushen Qinggan Formula is frequently used in clinical practice for treating hypertension, which is composed of* Gastrodia elata* (tian ma, TM) 30 g,* Uncaria* (gouteng, GT) 30 g,* Eucommia* bark (du zhong, DZ) 30 g, radix* Scutellariae* (huang qin, HQ) 15 g, and bitter butyl tea (kudingcha, KDC) 15 g. In Traditional Chinese Medicine (TCM) theory, most hypertensive patients show unbalance of Yin and Yang. There is a therapeutic strategy, namely, using “reinforcing” and “reducing” herbs together to balance Yin/Yang [18], and this strategy is used in the present study.

On the basis of previous, we hypothesize that BPV plays a role in the development of endothelial injury and leads to cardiovascular disease finally. Therefore, the aim of this clinical study is (1) to evaluate if the efficacy of Bushen Qinggan granule for blood pressure variability and endothelial injury in patients with hypertension is equivalent to that of the decoction and (2) to evaluate the difference in efficacy between the TCM formula and placebo.

## 2. Patients and Methods

This study was designed as a randomized, placebo-controlled study based on standard therapy and parallel groups. It was conducted in accordance with the Declaration of Helsinki. The study protocol was reviewed and approved by the Ethics Committee of Xiyuan Hospital of China Academy of Chinese Medical Sciences (2010XL016).

### 2.1. Patients

The target enrollment was 150 patients from the Cardiovascular Disease Clinic at the Xiyuan Hospital, the teaching hospital of China Academy of Chinese Medical Sciences. The enrollment criteria consisted of patients aged from 18 to 75 years and clinical findings of essential hypertension (EH) for at least 3 months prior to screening. Both men and women were included. Level 1 and level 2 hypertension was diagnosed in accordance with Chinese guidelines published in 2005 and 2010 for the management of hypertension [2, 19]. To be included in this trial, patients had to (1) have a 24-hour ambulatory blood pressure monitor (24 h ABPM): 24 h average blood pressure needs to ≥130/80 mm Hg, average blood pressure ≥135/85 mmHg during waking hours, or average blood pressure ≥120/70 mm Hg during sleeping hours, or (2) systolic blood pressure (SBP) ≥140 mmHg and/or diastolic blood pressure (DBP) ≥90 mm Hg, which was based on the average of 3 times seated BP readings or more office visits. Patients should have never used blood pressure medication or more than 3 times measured in one week reached the diagnostic criteria after elution. All included patients had signed written informed consent. Patients were excluded with secondary hypertension or hypertensive crisis. In addition, patients were excluded if they had uncorrected valvular heart disease, severe cardiac dysfunction, unstable angina, myocardial infarction, or stroke within half a year, had severe primary hepatic or renal disease, or had severe mental disorders or other uncontrolled systemic diseases. Finally, patients were excluded if females were pregnant or lactating, were known or suspected to be allergic to the study drugs, or had join other new drug clinical trials in recent 3 months.

From January 2010 to May 2012, participants were recruited from outpatients or via mailed brochures. Patients were evaluated based on physical examinations, laboratory screening, and ambulatory blood pressure monitor. Eligible patients were randomly assigned to 3 groups in a 1 : 1 : 1 ratio, who would have receive Bushen Qinggan granule, Bushen Qinggan decoction, or placebo in addition to a standard medication. A total of 150 patients who met the selection criteria were recruited. This study is designed as a pilot study and allowing for a 20% dropout rate. Participants will be assigned with a 1 : 1 : 1 allocation ratio according to a randomization list generated with an SAS software package. The allocation will be concealed in sequentially numbered, opaque, sealed envelopes. Each participant received an envelope upon recruitment sequence. The random allocation envelops will be opened only after the participant has satisfied all selection criteria and completed baseline assessments. The researchers will know the allocated group but the participants, outcome assessors, and statisticians will not. The dosage used in this study was one bag of Bushen Qinggan granule, decoction, or placebo 2 times daily. Patients attended follow-up appointments at the secondary, fourth, 6th, and 8th weeks of treatment. At each visit, patients were asked about the occurrence of any clinical event or adverse effect.

The study period lasted 8 weeks.

#### 2.1.1. Basic Interventions

The participants of all three groups used amlodipine 5 mg per day (Batch no. H10950224).

#### 2.1.2. Granule and Decoction Group

The Bushen Qinggan formula was taken orally by patients in both groups. The Bushen Qinggan formula was produced in the form of granules and decoction by Sichuan New Green Pharmaceutical Technology Development Co., LTD (Batch no. 100718206). Both granule and decoction were composed of Gastrodia elata (tianma, TM) 30 g,* Uncaria *(gouteng, GT) 30 g,* Eucommia *bark (du zhong, DZ) 30 g, radix* Scutellariae *(huang qin, HQ) 15 g, and bitter butyl tea (kudingcha, KDC) 15 g. Granules were dissolved in boiling water and drunk while still warm.

#### 2.1.3. Control Group

Placebo was taken by patients in the control group. The appearance, taste, and smelling of the placebo granules were made up to be extremely similar to the Bushen Qinggan granules. Instruction for usage was the same as Bushen Qinggan granules.

### 2.2. Endpoints

All patients were evaluated at baseline and weeks 2, 4, 6, and 8. The following clinical and laboratory variables were assessed at the first and last visit.

#### 2.2.1. Primary Endpoints

The primary endpoints were mean blood pressure and BPV, both measured by ambulatory blood pressure monitoring. We recorded 24-hour ambulatory blood pressure monitoring with 9027-ABP (SP(a) celabs Medica, USA). The cuff was programmed to inflate every 30 minutes between 6 a.m. and 10 p.m. (daytime) and every 60 minutes between 10 p.m. and 6 a.m. (night-time). The blood pressure data were edited by blood pressure data analysis software and processed by experienced analysts. 24-hour ambulatory blood pressure monitoring data were recorded at baseline and 8 weeks. There was no limit on activities during the monitoring.

Blood pressure variability (BPV) was defined as the standard deviation of mean blood pressure depending on its time period.

According to above definition, blood pressure data were collected both daytime (d) and nighttime (n) including SBP, DBP, SBP variability (SBPv), DBP variability (DBPv), SBP deviation (SBPd), and DBP deviation (DBPd) over 24 h.

#### 2.2.2. Secondary Endpoints

After at least 8 h of fasting, we collected venous blood from all participants at 8:00 a.m., for the test of endothelium related factors: endothelin-1 (ET-1, measured by nonequilibrium radioimmunoassay method; radiation immunoassay kits are provided by Beijing Huaying Biotechnology Research Institute; radioimmunoassay instrument is r-911 automatic counting device, produced by China University of Science and Technology Industrial Corporation), and nitric oxide (NO, is measured by colorimetric method, and the kit is provided by Beijing Huaying Biotechnology Research Institute; instrument manufacturer is Japan's Hitachi 7160 automatic biochemical analyzer).

#### 2.2.3. Assessment of Adverse Reactions

At each visit, patients were asked if there were any adverse effects. When an adverse event appeared, the timing relative to the administration of the drugs was noted. Blood cell count, electrolytes, serum creatinine, liver function test, urinalysis, and electrocardiogram results were recorded before and after treatment.

### 2.3. Statistical Analyses

Analyses were performed using SPSS for Windows, version 14.0 (SPSS, Chicago, IL). Data were described as mean ± standard deviation. Proportions were used to express dichotomous variables. One-way analysis of variance (ANOVA) test was used to compare the difference between three groups. Consistent with the requirement for ANOVA, all data were checked for variance homogeneity and for normal distribution. Data that were not normally distributed were analysed using Kruskal-Wallis *H* test. The paired *t*-test was used to compare data before and after treatment. All data were checked for normal distribution. Data that were not normally distributed were analysed using Wilcoxon rank sum test. Chi-square test was performed for dichotomous variables. For dropout and withdrawal patients, perprotocol (PP) analysis was adopted, considering one of the main purposes is to prove the equivalence of granule and decoction. *P* value of less than 0.05 was considered significant.

## 3. Results

**Figure 1:**
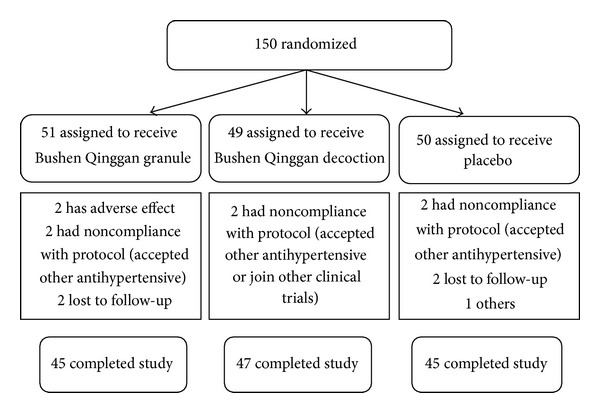
Flow diagram of patients in each group throughout the study.

A total of 137 patients (91.3%) completed the 8-week study, with premature termination occurring in 5 patients in the placebo group, 6 patients in the granule group, and 2 patients in the decoction group (see Figure 1).

### 3.1. Baseline Characteristics

There were 45 patients enrolled in the granule group (31 men and 14 women), and the average age was 49.93 ± 3.49 years old. There were 47 patients enrolled in the decoction group (33 men and 14 women), and the average age was 47.58 ± 5.02 years old. The control group had 45 cases (29 men and 16 women) with an average age of 48.34 ± 4.25 years old. The baseline characteristics were comparable and statistically insignificant among three groups (see Table 1).

### 3.2. Comparison of 24 h ABPM

Prior to interventions, there was no statistically significant difference in the blood pressure parameters measured by 24 h ABPM among three groups.

#### 3.2.1. Changes in Blood Pressure at Different Intervals before and after Treatment

In the control and two Bushen Qinggan groups, there was significant difference in blood pressure at different intervals before and after treatment (*P* < 0.01 or *P* < 0.05). There was significant decrease (*P* < 0.05) in the level of dSBP in the decoction group compared with the other groups (see Table 2).

#### 3.2.2. Changes in BPV at Different Intervals before and after Treatment

A favorable effect of Bushen Qinggan Formula was observed on control of BPV level after 8 weeks of treatment; both groups showed a significant decrease in BPV levels from baseline, but treatment with Bushen Qinggan Formula led to a significantly greater reduction than did the placebo There were significant decreases (*P* < 0.05) in the levels of 24-hSBPd, dSBPd, nSBPd, 24-hDBPv, 24-hDBPd, dDBPd, and nDBPd in the decoction and granule group compared with the control group; no significant differences were between granule and decoction group (*P* > 0.05) (see Table 3).

### 3.3. Changes in Endothelial Function

Compared with control group, granule and decoction group decreased ET-1 and elevated NO/ET-1 (*P* < 0.05); no significant differences were between granule and decoction group (*P* > 0.05) (see Table 4).

## 4. Discussion

Most scientific literature about comparison between granule and decoction focused on animal and cell experiments, including the animal experiment showing that Bushen Qinggan Formula improved NO in SHR (spontaneously hypertensive rat) [20]. According to the result, we suppose that Bushen Qinggan Formula trends to protect endothelial function of hypertension patients. Therefore, this study examined the integrative treatment with conventional drug therapy and Chinese herbal formula of Bushen Qinggan in hypertension patients. Compared with antihypertensive drugs alone, the integrative treatment exhibited better results in reducing BPV. At the same time, it effectively protected the function of endothelium. Bushen Qinggan Formula exerts multiple actions on hypertension patients and may therefore be an effective and efficient tool in the management of hypertension.

TCM practitioners treat patients based on certain syndromes of patients, that is, “Syndrome Differentiation and Treatment” or “Correspondence of Prescription and Syndrome.” Bushen Qinggan Formula is also guided by this concept. Use of the combination of 5 herbal medicines in Bushen Qinggan formulation for the antihypertensive effects was a common practice according to the experts in TCM. The use of Bushen Qinggan formulation highlights the combination of syndrome and disease which coincides with the concept and treatment of modern medicine [21].

A lot of clinical evidence shows that CM (Chinese Medicine) was effective for hypertension in clinical use [22], also possessing the advantage of the whole body regulation and TOD protection [23]. In recent years, pharmacologic studies also have confirmed that phenolic compounds and indole alkaloids contained in Gouteng have the antihypertensive, free radical scavenging, and antiexcitotoxic effects [24]. Huang qin [25] and tian ma [26] also have effects of being anti-inflammatory and antipyretic and may attribute to the protection of vascular endothelium. Furthermore, the vascular endothelium protective function also was approved in our research. Vascular health may influence the pathogenesis of cardiovascular disease, in part, through influences on BPV.

Currently, TCM granules are commonly used in the clinic, but the role and efficacy comparing with traditional decoction are still controversial. For this research, the efficacy of two different forms of Bushen Qinggan Formula is similar in improving BPV and endothelial function. This study is designed as a pilot clinical trial. From its results, we find the trends of equivalent conversion for the two formulations. However, the approximate effect of decoction and granules is noteworthy and deserving further study, in order to make more patients willing to accept TCM treatment.

Hypertension in three groups has no statistical difference; however, it is well controlled at the end of the study maybe because of the application of antihypertensive. These results suggest that blood pressure changes may not be the main function of the Chinese herb formula.

## Figures and Tables

**Table 1:** Baseline characteristics of three groups.

Variables	Granule group (*n* = 45)	Decoction group (*n* = 47)	Control group (*n* = 45)	*P* value
Age, y	49.93 ± 3.49	47.58 ± 5.02	48.34 ± 4.25	0.071
Men/women	31/14	33/14	29/16	0.382
Body mass index, kg/m^2^	26.75 ± 4.03	25.19 ± 4.87	25.46 ± 3.43	0.082
Stage of hypertension (cases)				
Stage I	20	23	22	0.242
Stage II	25	24	23	0.242
Previous cardiovascular diseases (cases)	8	6	7	0.448
Dyslipidemia, (cases)	9	11	8	0.455
Heart rates, bpm	75.66 ± 8.28	76.23 ± 8.99	75.94 ± 8.47	0.084

**Table 2:** Changes in blood pressure at different intervals before and after treatment in three groups (mean ± standard deviation, mmHg).

Index	Granule group (*n* = 45)		Decoction group (*n* = 47)		Control group (*n* = 45)	
	Before treatment	After treatment	Before treatment	After treatment	Before treatment	After treatment
24-hSBP	136.93 ± 12.49	129.22 ± 9.71∗	138.55 ± 11.02	127.57 ± 12.15∗∗	135.06 ± 13.25	130.37 ± 14.16∗
dSBP	147.75 ± 9.03	132.44 ± 10.55∗∗	144.19 ± 11.87	129.23 ± 12.28^∗∗△^	146.46 ± 7.43	133.35 ± 15.24∗
nSBP	127.66 ± 14.59	122.93 ± 12.91∗	125.49 ± 10.96	120.89 ± 13.31∗	127.60 ± 12.41	122.40 ± 13.01∗
24-hDBP	84.22 ± 9.89	77.60 ± 10.55∗	83.36 ± 10.69	78.38 ± 9.58∗	84.08 ± 8.92	79.84 ± 10.85∗
dDBP	86.48 ± 9.83	79.75 ± 11.12∗	85.49 ± 11.32	80.79 ± 10.56∗	86.44 ± 9.04	81.86 ± 11.40∗
nDBP	78.04 ± 10.70	71.13 ± 10.53∗	75.85 ± 10.43	73.17 ± 10.37∗	77.37 ± 9.58	72.82 ± 9.93∗

*P < 0.05, **P < 0.01, versus before treatment; ^△^ *P* < 0.05, versus control group. 24-hSBP: 24-hour systolic blood pressure; 24-hDBP: 24-hour diastolic blood pressure; dSBP: daytime systolic blood pressure; dDBP: daytime diastolic blood pressure; nSBP: night-time systolic blood pressure; nDBP: night-time diastolic blood pressure.

**Table 3:** Changes in blood pressure variability at different intervals before and after treatment in three groups (mean ± standard deviation, mmHg).

Index	Granule group (*n* = 45)		Decoction group (*n* = 47)		Control group (*n* = 45)	
	Before treatment	After treatment	Before treatment	After treatment	Before treatment	After treatment
24-hSBPd	15.68 ± 3.50	11.84 ± 1.93^∗∗△^	15.08 ± 4.89	11.57 ± 2.53^∗∗△^	15.41 ± 3.91	13.31 ± 3.03∗∗
24-hSBPv	11.43 ± 2.93	10.34 ± 2.19	10.60 ± 6.45	9.94 ± 2.12	11.02 ± 2.38	10.18 ± 2.28
dSBPd	13.17 ± 3.15	9.97 ± 2.34^∗∗△^	12.66 ± 3.20	9.96 ± 1.66^∗∗△^	13.68 ± 3.62	11.57 ± 2.74∗
dSBPv	8.92 ± 1.96	7.65 ± 2.55∗	9.16 ± 2.18	6.83 ± 4.83∗∗	9.33 ± 2.57	7.19 ± 3.69∗∗
nSBPd	11.68 ± 3.81	8.22 ± 2.42^∗△^	11.04 ± 3.40	8.21 ± 2.51^∗△^	12.42 ± 4.64	10.24 ± 4.27∗
nSBPv	8.92 ± 3.43	6.81 ± 2.60∗	8.76 ± 2.56	8.11 ± 3.57∗	9.03 ± 4.02	7.59 ± 4.46
24-hDBPd	10.53 ± 1.74	8.51 ± 1.54^∗∗△^	10.02 ± 2.63	8.50 ± 1.46^∗∗△^	11.26 ± 2.95	9.77 ± 1.69∗∗
24-hDBPv	12.78 ± 2.25	11.06 ± 2.09^∗∗△^	12.15 ± 3.25	10.24 ± 6.35^∗∗△^	13.20 ± 2.94	12.39 ± 2.28
dDBPd	8.84 ± 2.37	6.66 ± 1.53^∗∗△^	8.55 ± 2.29	6.89 ± 1.38^∗△^	9.33 ± 2.86	7.68 ± 1.75∗∗
dDBPv	10.35 ± 2.76	8.36 ± 2.83∗∗	10.58 ± 3.26	7.86 ± 4.94∗∗	10.63 ± 3.05	8.25 ± 3.58∗∗
nDBPd	8.95 ± 3.06	6.57 ± 1.87^∗∗△^	8.98 ± 2.87	6.79 ± 2.21^∗∗△^	9.66 ± 3.46	8.11 ± 3.20∗
nDBPv	11.35 ± 4.64	8.94 ± 3.39∗∗	10.32 ± 6.94	7.19 ± 5.29∗	10.98 ± 5.47	10.15 ± 5.41

*P < 0.05, **P < 0.01, versus before treatment; ^△^ *P* < 0.05, versus control group. 24-hSBPd: 24-hour systolic blood pressure deviation; 24-hSBPv: 24-hour systolic blood pressure variability; dSBPd: daytime systolic blood pressure deviation; dSBPv: daytime systolic blood pressure variability; nSBPd: night-time systolic blood pressure deviation; nSBPv: night-time systolic blood pressure variability; 24-hDBPd: 24-hour diastolic blood pressure deviation; 24-hDBPv: 24-hour diastolic blood pressure variability; dDBPd: daytime diastolic blood pressure deviation; dDBPv: daytime diastolic blood pressure variability; nDBPd: night-time diastolic blood pressure deviation; nDBPv: night-time diastolic blood pressure variability.

**Table 4:** Changes inendothelial function before and after treatment in three groups (mean ± standard deviation).

Index	Granule group (*n* = 45)		Decoction group (*n* = 47)		Control group (*n* = 45)	
	Before treatment	After treatment	Before treatment	After treatment	Before treatment	After treatment
NO (*μ*mol/L)	56.61 ± 15.30	62.63 ± 15.51	56.77 ± 9.60	62.81 ± 8.69	57.86 ± 13.77	58.98 ± 14.47
ET-1 (pg/mL)	66.26 ± 9.20	59.97 ± 6.21^∗△^	65.05 ± 13.77	59.91 ± 10.41^∗△^	64.37 ± 8.88	64.71 ± 9.35
NO/ET	0.87 ± 0.30	1.05 ± 0.28^∗∗△^	0.95 ± 0.52	1.08 ± 0.24^∗∗△^	0.92 ± 0.28	0.93 ± 0.27

*P < 0.05, **P < 0.01, versus before treatment; ^△^ *P* < 0.05, versus control group.
